# Medical Student Experiences With ChatGPT: National Cross-Sectional Study

**DOI:** 10.2196/76838

**Published:** 2026-03-09

**Authors:** Alan Yuesheng Xu, Skye Speakman, Vincent Salvatore Piranio, Robert Medina, Michelle Liu, Chris Lamprecht, Nicolas Abchee, Meghan Brennan

**Affiliations:** 1 College of Medicine University of Florida Gainesville, FL United States

**Keywords:** ChatGPT, large language model, medical student, medical education, artificial intelligence, AI

## Abstract

**Background:**

Artificial intelligence (AI) is increasingly influencing medical student education, with AI-driven chatbots, such as ChatGPT, emerging as powerful study tools. While these technologies offer numerous benefits, they also pose challenges that warrant the adaptation of medical school curricula.

**Objective:**

This study examines medical students’ perceptions and use of ChatGPT. We hypothesize that ChatGPT is widely used for academic support, but concerns remain regarding reliability and academic integrity.

**Methods:**

We conducted a cross-sectional study from August 25 to December 10, 2024, in the United States. Students in all years of medical training who were enrolled in accredited allopathic or osteopathic medical schools were eligible to participate. Data were collected using an anonymous online questionnaire, which was distributed through institutional mailing lists. Overall, 188 schools were reached, of which 14 (7.4%) responded and agreed to distribute the survey. A total of 177 participants completed the survey. Survey items consisted primarily of Likert-scale and multiple-choice questions. Primary outcome measures included self-reported frequency of ChatGPT use, perceived usefulness of ChatGPT, and ChatGPT use habits.

**Results:**

Overall, 98.9% (175/177) of participants had heard of ChatGPT, with 88.7% (157/177) reporting having used it; 62.7% (111/177) identified as female, and 52% (92/177) had completed at least 1 block of clinical rotations. Medical students most often used ChatGPT to understand complex medical concepts, prepare for exams, and generate study materials. Moreover, 46.5% (73/157) used it to help complete medical school assignments. Medical students also reported using it clinically, with the most common use being to generate differential diagnoses. Notably, 21.0% (33/157) of participants responded having used ChatGPT to help write clinical notes. Moreover, 73.9% (116/157) reported that their experience with ChatGPT improved their overall perception of AI’s potential to assist in medical practice, and 86.6% (135/157) believed that having ChatGPT as a resource would make them more effective physicians. Statistical analyses were performed using the Pearson chi-square test with α=.05. Students who reported moderate or advanced baseline understanding of AI were more likely to practice conscientious use habits, such as cross-checking (odds ratio [OR] 2.31, 95% CI 1.08-4.97) and editing (OR 2.45, 95% CI 1.05-5.71) ChatGPT output before using it, than those who reported a basic or limited understanding.

**Conclusions:**

Our study is among the few to examine medical student perceptions of ChatGPT at a national level. We examined responsible use habits to identify areas in which reliance on this technology may lead users astray. We found that ChatGPT is being used to complete academic assignments and write clinical notes, raising concerns about information verification, AI literacy, patient confidentiality, and ethical use. Together, these findings highlight the need for structured AI education to help students leverage these technologies effectively while mitigating risks associated with misinformation and overreliance on AI.

## Introduction

### Problem

Artificial intelligence (AI) is progressing rapidly, with increasing applications in health care, including medical education. Among AI-driven technologies, chatbots powered by large language models (LLMs) have gained significant attention for their potential to support learning, clinical decision-making, and research. These chatbots can generate humanlike responses based on complex pattern recognition and statistical synthesis of massive datasets comprising papers, books, websites, and other written text [1]. Perhaps the most prominent example of such technology is ChatGPT, an LLM-based chatbot that was launched by OpenAI in November 2022.

ChatGPT was likely one of the fastest-growing user applications in internet history, with nearly 100 million users as of January 2023 and approximately 1.8 billion website visitors monthly [2]. In the context of the medical field, it is reasonable to expect that both medical providers and patients have interfaced with and used such technology. ChatGPT and other LLMs have already influenced medical education, providing medical students with a powerful tool for education while also raising new issues regarding academic dishonesty, plagiarism, and ethics [3].

As medical students seek efficient ways to enhance their education, the integration of AI-powered tools raises important questions about their usefulness, reliability, and impact on learning. Understanding medical students’ perceptions of these technologies is crucial for assessing their role in medical training and future clinical practice. Moreover, this integration raises important questions about the necessity of adapting medical curricula in the United States to address the increasing use of AI, as well as to establish policies regarding academic integrity and the ethical use of AI in patient care to protect patient confidentiality and critical thinking skills.

### Review of Relevant Scholarship

There have been few studies examining medical student perceptions and use of AI chatbots, such as ChatGPT; moreover, many of these studies were conducted at single institutions or overseas. A single-institution study in Thailand found that medical students had begun using AI-based chatbots with generally positive perceptions [4]. A 2023 cross-sectional study in Germany, Austria, and Switzerland found that more than one-third of medical students had already used an AI-based chatbot, such as ChatGPT, but the majority had not been exposed to formal AI or AI ethics curricula [5]. However, there have been few studies examining the use habits associated with such technology and the resulting ethical concerns. A cross-sectional study in Jordan found that younger students and those with lower AI proficiency used ChatGPT more frequently [6]. In our previous single-institution study of medical students’ use of LLM-based chatbots, we found that medical students were already integrating these technologies into their academics and even patient care [7]. Moreover, having a baseline exposure or knowledge of AI was associated with more conscientious technology use habits such as double-checking information obtained [7]. A 2023 cross-sectional study in the United States found that medical students identified HIPAA (Health Insurance Portability and Accountability Act)–compliance concerns with ChatGPT and endorsed clearer guidelines for its use [8]. Despite this, many medical schools are still unprepared, with a recent survey of US osteopathic medical schools finding that many lacked formal policies for the integration of generative AI [9].

### Hypothesis, Aims, and Objectives

In this study, we examined perceptions of ChatGPT across various medical schools spanning different geographic locations nationwide. The goal of our study was to explore how current medical students are using ChatGPT on a national level and to identify what students find most useful for augmenting learning and productivity. Importantly, this study aimed to identify pitfalls or risks of use as well as opportunities for targeted educational support to improve medical education. On the basis of previous research, we hypothesize that ChatGPT is widely used among students primarily for academic study support and writing tasks, but concerns remain regarding reliability and academic integrity.

## Methods

### Inclusion and Exclusion Criteria

Students in all years of medical training in the United States who were enrolled in either accredited allopathic or osteopathic medical schools during the study period were eligible to participate.

### Sampling Procedure and Recruitment

We used a nonprobability referral sampling design, recruiting participants through institutional mailing lists distributed by deans of student affairs and student representatives at US medical schools.

Email addresses of the deans of student affairs were collected by reviewing publicly available information on medical school websites. One senior coauthor subsequently sent out email requests to the deans to survey their respective medical student populations, reaching a total of 188 allopathic and osteopathic medical schools in the United States from August 25, 2024, to December 10, 2024. If permission was granted by the dean, a link to the online survey was distributed to their schools’ respective mailing lists of currently enrolled medical students across all years of training. If no response was received from the deans, a follow-up email was sent 1 week later by the same senior coauthor.

One senior coauthor also contacted members from the Organization of Student Representatives using social media to similarly distribute the survey link to their student bodies via email listserv. Participants were given a 4-week deadline from the date the survey link was distributed to complete the survey. The institutions included in this study were those that responded and agreed to distribute the survey to the email addresses of their medical students. A total of 14 schools responded and agreed to participate.

### Survey Design

The open survey consisted of 28 questions (Multimedia Appendix 1). Participants were queried on their use of ChatGPT in the domains of academic use, research, and patient care. Participants were also queried, with Likert-scale response options, about their perception of ChatGPT and about AI in general. The final section of the survey collected demographic information. Those who responded that they had not heard of or used ChatGPT were automatically directed to the end of the survey, which contained the AI perception and demographic sections. The survey was developed from a single-institution pilot study. In addition, it was piloted among 7 students to test the functionality of the survey and to obtain a time-to-completion estimate for survey distribution.

### Instrumentation

The online questionnaire was administered using Qualtrics (Qualtrics LLC), a secure web-based survey platform. Cookies and IP addresses were automatically detected by Qualtrics to prevent duplicate submissions. Overall, the online questionnaire was designed and distributed in accordance with the CHERRIES (Checklist for Reporting Results of Internet E-Surveys) checklist [10]. This manuscript was written and organized according to the JARS quantitative reporting standards [11].

### Data Diagnostics

Likert-scale categories showed complete separation of data in many instances, so we collapsed the items into binary indicators to permit stable model estimation. Some response variable combinations contained zero observations or very few observations (<5), which did not permit other forms of regression models. For questions in which answer choices were reported as frequencies (0%, 25%, 50%, 75%, and 100%), frequencies of 50% and below were considered infrequent and reclassified as such, whereas those above 50% were classified as “frequent.” We dichotomized at this point because it provided a straightforward, practical interpretation. Given the data separation, higher-order Likert categories could not be modeled. Incomplete responses referred to questions in which one or more items were left unanswered. Although these surveys were retained, only completed cases were included for statistical analysis. Missing data were not imputed, as all data summaries and analyses were conducted using only observed responses.

### Analytic Strategy

Statistical analyses were conducted using JMP Pro (version 18; JMP Statistical Discovery LLC), with the significance level set at *P*<.05. The Pearson chi-square test was used to determine associations between survey responses.

### Ethical Considerations

This study was determined to be exempt from review by the University of Florida Institutional Review Board (ET00042341) because it involved an anonymous online survey with minimal risk to participants. However, all participants were presented with a one-page description of the study before participating, including the purpose of the study, time required, research benefits, risks, confidentiality, data storage procedures, contact information of the principal investigator, and notice of voluntary participation. The study was anonymous, and no personal information was collected from the Qualtrics platform; however, it was up to the participants’ discretion to list which medical school they attended. No identifying data were collected or presented in this manuscript. Participants were not compensated, and participation was completely voluntary.

## Results

### User Statistics

A total of 188 schools were contacted, of which 14 (7.4%) responded and agreed to distribute the survey. There were 228 total responses, of which 177 (77.6%) were completed survey responses. While the true response rate is unknown, we conservatively estimate a response rate of 2.3% (228/10,017), with the denominator based on publicly available data on the total class sizes of the schools that participated in the survey. The following schools were represented:

- Carle Illinois College of Medicine
- Kansas City University College of Osteopathic Medicine
- The Joe R & Teresa Lozano Long School of Medicine
- NYU Grossman School of Medicine
- Perelman School of Medicine at the University of Pennsylvania
- The Texas College of Osteopathic Medicine
- The Warren Alpert Medical School of Brown University
- The University of Florida College of Medicine
- The University of the Incarnate Word School of Osteopathic Medicine
- The University of Central Florida College of Medicine
- Wayne State University School of Medicine
- The Western Michigan University Homer Stryker MD School of Medicine
- Western University College of Osteopathic Medicine of the Pacific
- William Carey University College of Osteopathic Medicine

In total, 98.9% (175/177) of the participants responded that they had heard of ChatGPT, and 88.7% (157/177) reported having ever used ChatGPT. The demographics of respondents are shown in Table 1. Moreover, 27.7% (49/177) of respondents reported having only a limited or basic understanding of AI, whereas the majority (128/177, 72.3%) reported having a moderate or higher understanding (Table 2). Additionally, 31.6% (56/177) of respondents reported having been exposed to AI integration or education in their medical curriculum.

**Table 1:** Self-reported demographics of participants^a^.

Demographics		Medical students, n (%)
**Age (years)**		
	<20	0 (0.0)
	20-24	62 (35.0)
	25-30	101 (57.1)
	>30	14 (7.9)
**Sex**		
	Female	111 (62.7)
	Male	63 (35.6)
	Other	1 (0.6)
	Prefer not to answer	2 (1.1)
**How do you self-identify?**		
	American Indian or Alaska Native	4 (2.3)
	Asian	40 (22.6)
	Black or African American	10 (5.6)
	Hispanic, Latino, or Spanish origin	14 (7.9)
	Native Hawaiian or other Pacific Islander	1 (0.6)
	White	95 (53.7)
	Other	8 (4.5)
	Prefer not to answer	5 (2.8)
**Year of medical school**		
	First	51 (28.8)
	Second	46 (26.0)
	Third	34 (19.2)
	Fourth	44 (24.9)
	Fifth or beyond	2 (1.1)
**Completed at least 1 block of clinical rotations**		
	No	92 (52.0)
	Yes	85 (48.0)

^a^Cross-sectional study of medical student perceptions of ChatGPT in the United States from August 25, 2024, to December 10, 2024.

**Table 2:** Self-reported baseline understanding of artificial intelligence (AI) among participants^a^.

How would you describe your current understanding of AI?	Medical students, n (%)
No understanding: “I have never heard of AI.”	0 (0.0)
Limited understanding: “I have heard of the terminology but do not understand how it works.”	6 (3.4)
Basic understanding: “I understand that AI involves machines performing tasks that typically require human intelligence, but I am not familiar with its applications.”	43 (24.3)
Moderate understanding: “I have a general understanding of the key principles of AI and its common applications.”	119 (67.2)
Advanced understanding: “I possess a comprehensive understanding of AI principles, including deep learning, neural networks, and their complex implementations in real-world scenarios.”	9 (5.1)

^a^Cross-sectional study of medical student perceptions of ChatGPT in the United States from August 25, 2024, to December 10, 2024.

### Statistics and Data Analysis

**Figure 1:**
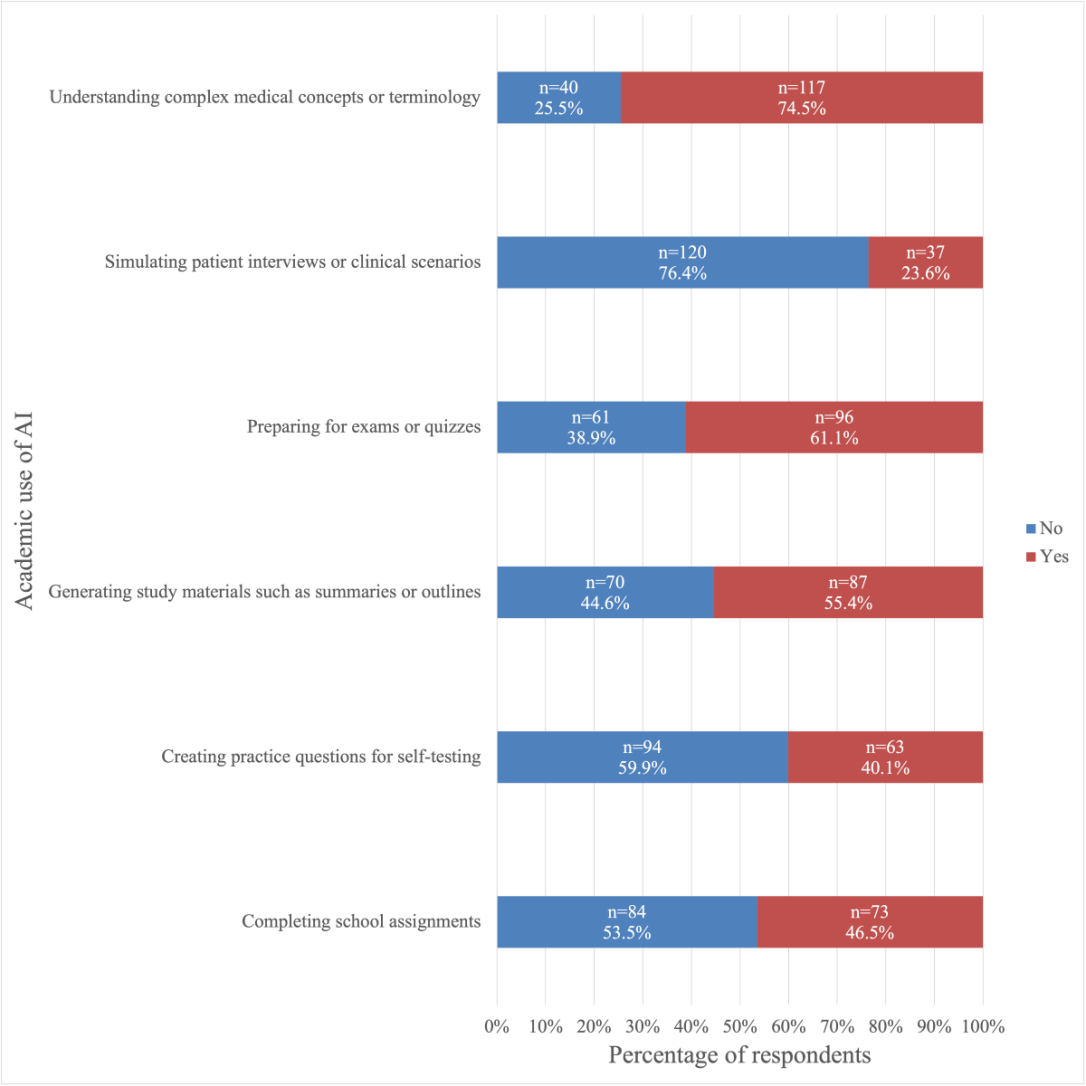
Frequency of use of ChatGPT among medical students in the United States (August 25 to December 10, 2024) for academic-related purposes in medical school. AI: artificial intelligence.

**Figure 2:**
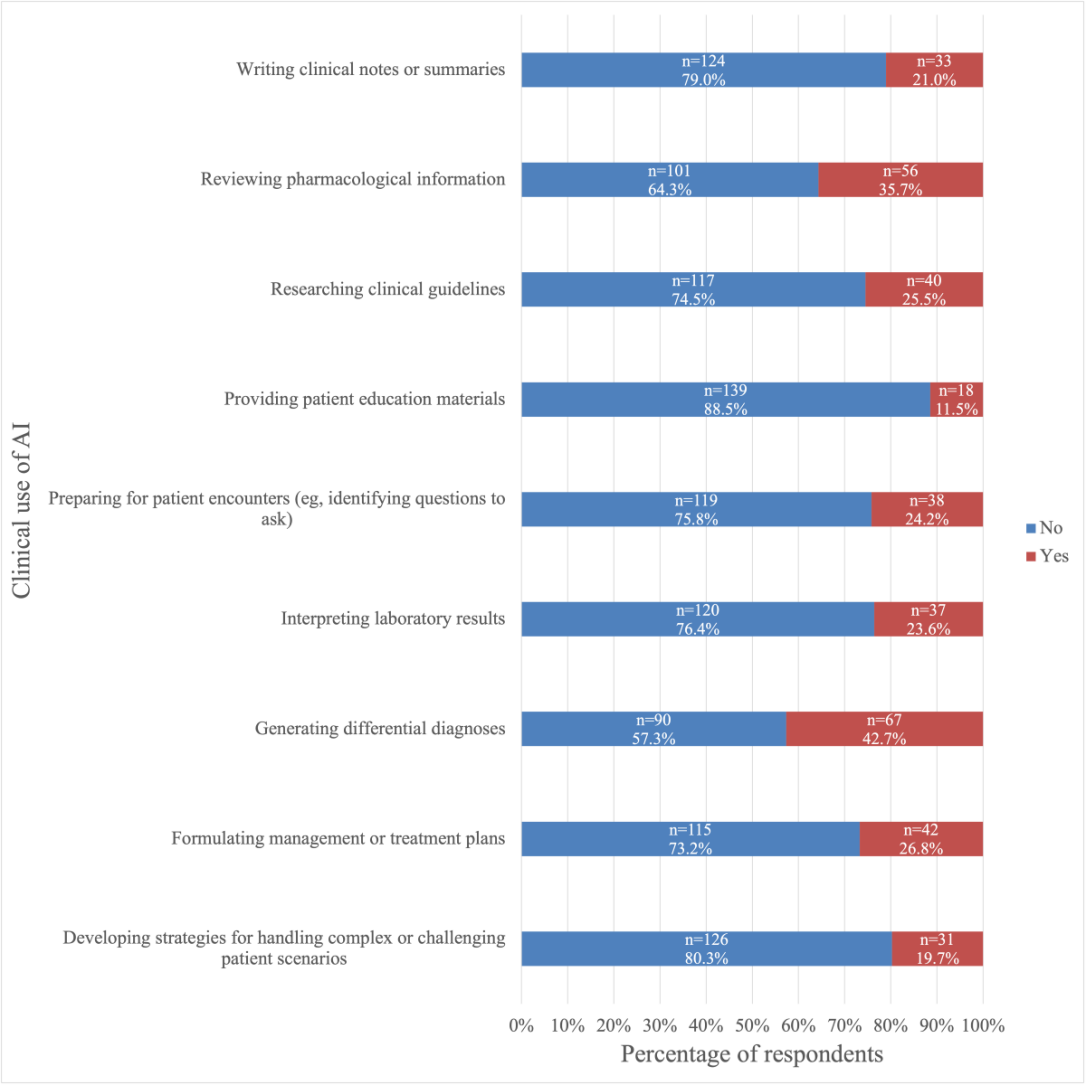
Frequency of use of ChatGPT among medical students in the United States (August 25 to December 10, 2024) for clinical-related purposes in medical school. AI: artificial intelligence.

**Figure 3:**
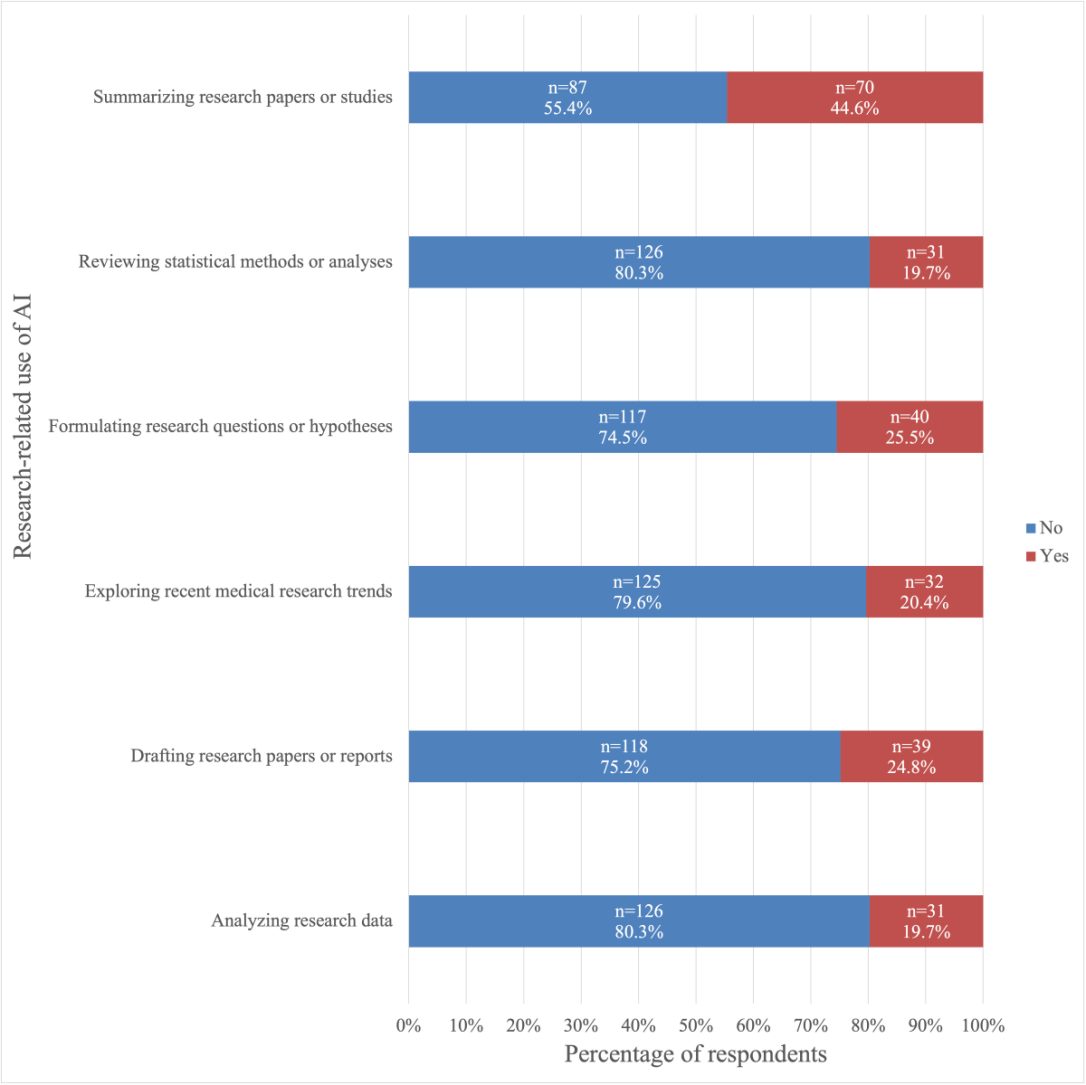
Frequency of use of ChatGPT among medical students in the United States (August 25 to December 10, 2024) for research-related purposes in medical school. AI: artificial intelligence.

We examined whether participants had used ChatGPT for academic, clinical, or research purposes. For each category, we developed specific use cases that were representative of each educational area and asked participants to indicate whether they had used the tool for those purposes. The results are shown in Figures 1-3.

Among students who used ChatGPT for medical school-related purposes, the most common academic use cases were helping understand complex medical concepts or terminology (117/157, 74.5%), followed by preparing for exams or quizzes (96/157, 61.1%), and generating study materials such as summaries or outlines (87/157, 55.4%; Figure 1). Additionally, 46.5% (73/157) reported having used ChatGPT to help complete medical school assignments (Figure 1). The most common clinical use cases of ChatGPT were generating differential diagnoses (67/157, 42.7%) and reviewing pharmacological information (56/157, 35.7%; Figure 2). Furthermore, 21.0% (33/157) had used it to help write clinical notes (Figure 2). Moreover, 57.6% (19/33) of these students reported that they were in the first or second year of medical school. The most common research-related use case was summarizing research papers or studies (70/157, 44.6%; Figure 3). Additionally, 24.8% (39/157) had used it to draft research manuscripts (Figure 3).

Participants were queried on their perception of the accuracy and usefulness of ChatGPT. In total, 75.2% (118/157) of respondents reported that the information provided by ChatGPT was more accurate than not, and 90.4% (142/157) agreed that ChatGPT’s responses were useful for their medical-related queries (Table 3). Moreover, 73.9% (116/157) reported that their experience with ChatGPT improved their overall perception of AI’s potential to assist in medical practice, and 86.6% (136/157) believed that having ChatGPT as a resource would help them better manage patient care and make them more effective physicians (Table 3). Additionally, 86.0% (135/157) believed that they would continue to use ChatGPT or similar AI chatbots in their future medical practice.

**Table 3:** Participants’ overall perceptions of ChatGPT for medical-related purposes^a^.

Questions		Medical students, n (%)
**When using ChatGPT for medical-related queries, how would you rate the accuracy of the information given?**		
	Very inaccurate: consistently gives incorrect information	0 (0.0)
	Inaccurate: more often gives incorrect information	1 (0.6)
	Somewhat inaccurate or accurate: gives a similar frequency of incorrect and correct information	29 (18.5)
	Accurate: more often gives correct information	97 (61.8)
	Very accurate: consistently gives correct information	21 (13.4)
	N/A^b^: “I have not used ChatGPT in this context.”	9 (5.7)
**Overall, the responses provided by ChatGPT were useful for your medical school–related queries.**		
	Strongly disagree	2 (1.3)
	Disagree	4 (2.6)
	Agree	88 (56.0)
	Strongly agree	54 (34.4)
	N/A: “I have not used ChatGPT in this context.”	9 (5.7)
**How has your experience with ChatGPT influenced your overall perception of AI’s potential to assist in medical practice?**		
	Somewhat worsened	8 (5.1)
	No change	33 (21.0)
	Somewhat improved	82 (52.2)
	Significantly improved	34 (21.7)
**To what extent do you agree that having ChatGPT as a resource would help you better manage patient care and make you a more effective physician?**		
	Strongly disagree	2 (1.3)
	Disagree	19 (12.1)
	Agree	98 (62.4)
	Strongly agree	38 (24.2)

^a^Cross-sectional study of medical student perceptions of ChatGPT in the United States from August 25, 2024, to December 10, 2024.

^b^N/A: not applicable.

Respondents were asked if they verified the accuracy of ChatGPT responses and how often they did so. In total, 46.5% (73/157) of students reported cross-checking or double-checking the responses they received 50% of the time or less (Table 4). Moreover, 21.0% (33/157) reported editing or refining ChatGPT’s output before using it 50% of the time or less (Table 4).

**Table 4:** Participant responses regarding ChatGPT use habits for medical purposes^a^.

Questions		Medical students, n (%)
**When using ChatGPT for medical school–related queries, how often do you cross-check or double-check the responses you receive?**		
	Almost never: about 0% of the time	9 (5.7)
	Occasionally: about 25% of the time	24 (15.3)
	Sometimes: about 50% of the time	42 (26.8)
	Frequently: about 75% of the time	40 (25.5)
	Almost always: almost 100% of the time	31 (19.8)
	N/A^b^: “I have not used ChatGPT in this context.”	11 (7.0)
**When using ChatGPT for medical school–related purposes, how often do you edit or refine ChatGPT’s output before using it?**		
	Almost never: about 0% of the time	8 (5.1)
	Occasionally: about 25% of the time	8 (5.1)
	Sometimes: about 50% of the time	17 (10.8)
	Frequently: about 75% of the time	30 (19.1)
	Almost always: almost 100% of the time	66 (42.0)
	N/A: “I have not used ChatGPT in this context.”	28 (17.8)

^a^Cross-sectional study of medical student perceptions of ChatGPT in the United States from August 25, 2024, to December 10, 2024.

^b^N/A: not applicable.

We conducted a chi-square analysis to examine the association between baseline understanding of AI and frequency of cross-checking and editing information obtained from ChatGPT. “Limited” and “basic” understanding of AI were categorized into one group, and “moderate” and “advanced” understanding were categorized into another. Frequencies of 50% or less were categorized as “infrequent,” and those above 50% were categorized as “frequent.” We found that those with moderate or advanced baseline understanding of AI were more likely to cross-check (odds ratio [OR] 2.31, 95% CI 1.08-4.97) and more likely to edit the output (OR 2.45, 95% CI 1.05-5.71) from ChatGPT before using it than those with a basic or limited understanding (Table 5).

**Table 5:** Statistical analysis of participant baseline understanding of artificial intelligence (AI) and the likelihood of responsible AI use habits^a^.

Responsible AI use habit	*P* value	Odds ratio (95% CI)
Moderate or advanced baseline understanding of AI and the frequency of cross-checking information obtained from ChatGPT	.03	2.31 (1.08-4.97)
Moderate or advanced baseline understanding of AI and the frequency of editing output from ChatGPT	.04	2.45 (1.05-5.71)

^a^Pearson chi-square was performed with an α=.05. Data were dichotomized to compare those with self-reported moderate or advanced baseline understanding of AI vs basic or limited understanding. Cross-sectional study of medical student perceptions of ChatGPT in the United States from August 25, 2024, to December 10, 2024.

**Figure 4:**
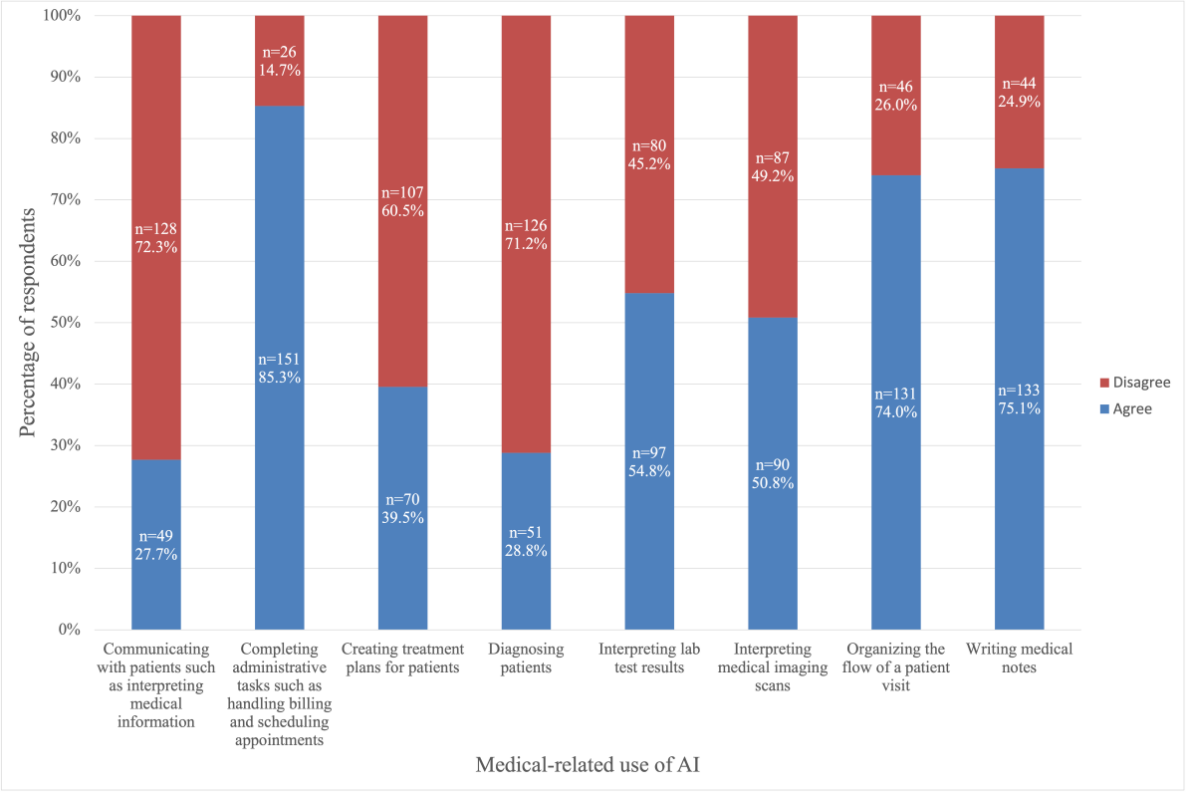
Participant comfort with artificial intelligence (AI) performing various medical-related tasks. Cross-sectional study of medical student perceptions of ChatGPT in the United States from August 25 to December 10, 2024.

We queried participants on their perception of AI in health care and medical education. In total, 80.2% (142/177) of the respondents believed that concepts of AI should be taught in medical school. Respondents were most comfortable with AI assisting in completing administrative tasks such as handling billing and scheduling appointments (151/177, 85.3%), writing medical notes (133/177, 75.1%), and organizing the flow of a patient visit (131/177, 74.0%). However, respondents were less comfortable with AI assisting in diagnosis and management decision-making (Figure 4).

**Figure 5:**
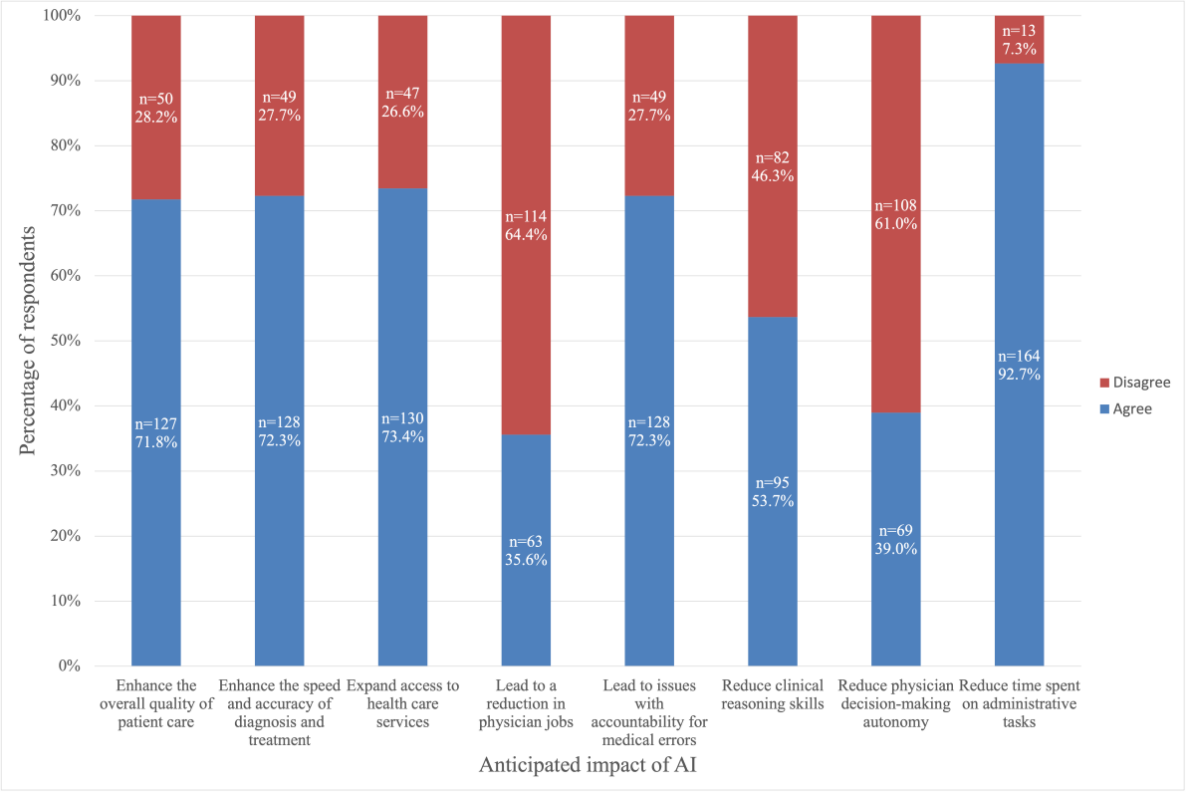
Participant opinions on artificial intelligences (AI) future impact on health care. Cross-sectional study of medical student perceptions of ChatGPT in the United States from August 25 to December 10, 2024.

Moreover, we surveyed medical students’ overall outlook of AI’s impact on health care. Most respondents believed that AI would reduce time spent on administrative tasks, expand access to health care services, enhance the overall quality of patient care, and improve the speed and accuracy of diagnoses and treatments. However, 35.6% (63/177) of participants believed it would lead to a reduction in physician jobs, 53.7% (95/177) believed it would reduce clinical reasoning skills, and 39.0% (69/177) believed it would reduce physician decision-making autonomy (Figure 5).

## Discussion

### Support of Original Hypotheses

Our findings support the hypothesis that medical students across the United States are actively incorporating ChatGPT as a support tool in medical education. Our previous single-institution study found that students were frequently using ChatGPT for clinical, academic, and research purposes [7]. This study further corroborates those results and elucidates specific use cases within these categories. Moreover, our findings suggest that there are concerns around the ethical and safe use of such technology, with students who have higher baseline AI knowledge being more likely to practice conscientious AI use habits. Overall, these findings warrant additional educational and policy interventions.

### Interpretation

Previous literature has highlighted the hypothetical utility of LLM-based tools in augmenting academic writing, generating practice questions with detailed answers, creating patient-physician simulations, suggesting new ideas for research, and creating personalized study materials [12,13]. Our results demonstrate that students are already using ChatGPT for these purposes. Although less common than academic applications, students also used ChatGPT in clinical practice, most frequently to generate differential diagnoses. Studies indicate a high rate of correct diagnosis within the differential list generated by ChatGPT. One study reported an accuracy exceeding 80% with ChatGPT [14]. Another study evaluating its accuracy using clinical notes recorded within the first 24 hours of emergency department admission found that ChatGPT had high accuracy in diagnosing common presentations but lower accuracy in diagnosing infrequent conditions or conditions with subtler clinical presentation, likely due to poorer representation in training data [15].

However, the clinical utility that LLM-based tools bring to the table may extend beyond merely providing a correct diagnosis. Another study suggested that it may increase confidence in diagnosis by bringing up other potential differential diagnoses or offering suggestions similar to those of specialists, which may help diagnostic workflow in lower-resource settings [16]. In certain institutions with student-run clinics for underinsured or uninsured patients, this may directly translate to improved patient care. Overall, this feature of LLM-based tools such as ChatGPT may help medical students perform better on clinical rotations by performing more comprehensive histories and assessments and developing more thorough plans.

Respondents also generally had a positive attitude toward ChatGPT. The majority reported that the information reported by ChatGPT was accurate and agreed that it was useful for their medical-related queries. Respondents indicated that they were likely to continue using ChatGPT or similar tools in their future medical practice (ie, as resident or attending physicians). Respondents also reported that their experience with ChatGPT improved their overall perception of AI’s potential to assist in medical practice. Many also responded that having ChatGPT as a resource would facilitate better patient care and enhance their effectiveness as physicians, further emphasizing the probable continued integration of AI technology into medical practice.

However, there are emerging challenges related to ensuring academic integrity, stopping the spread of misinformation, and preventing the erosion of independent clinical reasoning skills with the more widespread adoption of this technology [12]. We found that almost half of the respondents had used ChatGPT to help complete medical school assignments. Owing to ChatGPT’s access to a huge knowledge base and its ability to generate novel and detailed content, it is not surprising that students may use this tool to help answer open-ended questions in assignments or draft essays. This raises concerns about students relying on AI-generated text without critically engaging with the material. If students increasingly use ChatGPT as a shortcut rather than a supplementary tool, it risks diminishing analytic skills, weakening foundational medical knowledge, and promoting plagiarism.

There are also patient privacy and safety concerns associated with inputting sensitive patient information into ChatGPT. Whether using ChatGPT to generate a differential diagnosis list or to formulate a management plan, medical students and physicians must take caution to deidentify data and protect patient information. Respondents were not asked about the degree to which the information to input was deidentified; however, some respondents had reported using ChatGPT for writing clinical notes. Notably, many of these students who answered “yes” reported that they were in the first or second year of medical school, indicating that it may have been perceived as a theoretical use, given that most clinical rotations begin in years 3 and 4. It is also important to note that when this survey was sent out, there was little guidance or formal educational policies regarding the ethical use of AI in clinical practice. Nevertheless, this finding does present an opportunity for more formal education for both medical students and physicians alike on the importance of deidentifying patient data for future AI technologies.

Additionally, ChatGPT’s potential to generate inaccurate or misleading information means that students who do not critically evaluate its outputs may internalize incorrect medical knowledge. Although we did not explicitly ask our participants this, it is possible that ChatGPT could be used to answer multiple-choice or open-ended questions on take-home quizzes and examinations. This poses a challenge for medical education institutions to adapt their academic policies and assessment methods to ensure that students develop essential critical thinking skills and problem-solving skills.

Another issue is the well-known tendency of LLM-based chatbots to fabricate data, or “hallucinate,” in a manner where the generated responses appear convincing and correct, which can lead to medical error if users do not cross-check the information or exercise their own clinical judgment [17-20]. The latter is more likely to affect those with less experience, such as medical students or junior physicians. As such, it is crucial to adopt a conscientious approach to the use of these AI tools and for educational systems to deliberately engage future physicians in this practice early in training. We found that many participants infrequently cross-checked or double-checked the responses or edited the output before using it. Infrequency was defined as double-checking or editing 50% or less of the time. We did not specifically ask participants to identify or define what resources students use to cross-check information; while this may reflect the superior accuracy and quality of the output provided by ChatGPT, we used it as a metric of awareness of the need to critically evaluate AI-provided information, acknowledging its fallibility. We found that those who reported a stronger baseline understanding of AI were more likely to double-check and refine the output from ChatGPT before using it. This likely reflects a greater appreciation for the limitations of these AI tools, and such practice should be encouraged in the future to avoid medical error and underreliance on clinical judgment. This further emphasizes the need, at an institutional level, to educate future physicians about the limitations of AI tools they will inevitably encounter in clinical practice to ensure their most effective and responsible use. The quality of the output of LLM-based chatbots is also highly dependent on the user input or prompt provided. “Prompt engineering,” which involves thoughtfully designing prompts to optimize and tailor the output of AI tools, may become an integral part of medical education as we teach future physicians how to get the most out of their AI tools.

### Similarity of Results

Several survey studies from international medical institutions have examined medical students’ perception of LLM-based chatbots. A 2023 cross-sectional study conducted among graduated medical students at a medical center in the United Arab Emirates found that most students had not begun using ChatGPT during medical school at that time [21]. However, previous AI experience was strongly linked to a positive perception of AI’s role in patient care, reducing medical errors, and improving diagnostic accuracy [21]. Another cross-sectional study at the Faculty of Medicine of the University of Porto found that 16% of students were using ChatGPT daily, with 92% expressing satisfaction with the responses generated [22]. We previously conducted a pilot study of medical students’ perceptions of LLM chatbots locally at our institution, the University of Florida. We found that 69% of respondents had already been using LLM chatbots, such as ChatGPT, for medical-related purposes at least once a month or more [7]. We also found that those who had a baseline understanding of AI and exposure to AI in medical school were more likely to practice conscientious use of these chatbots, such as cross-checking the information obtained [7]. Those who were exposed to AI in medical school were also more likely to trust AI with clinical decision-making [7]. Our results are consistent with cross-sectional studies performed at other institutions, in which students overall demonstrate positive attitudes and an optimistic outlook toward LLM-based tools and AI in general. However, our study adds important insights regarding the ethical and safe use of AI technologies, underscoring the need for formal generative AI policies in medical schools and, potentially, the development of a dedicated AI curriculum.

### Limitations

A limitation of this study is the low survey response rate, which limits the generalizability of the survey results. Low response rate in our survey may be related to the length or complexity of the survey, as it included 28 questions, as well as competing time demands during medical education and a lack of perceived benefit from completing the survey. Additionally, email surveys may have lower response rates than more traditional methods, and given the abundance of phishing attempts, there may have been a reluctance to open an email from an unfamiliar sender. Low response rate among medical learners in general is common, given the large number of medical learners in US medical schools [23]. In addition, there is an increasing survey burnout among medical students, and there is a trend to limit the number of surveys medical students receive, which may have affected the delivery mode and response rate [24]. Despite the low response rate, our sample was diverse, with broad geographic representation and participation from both osteopathic and allopathic institutions.

Our survey was voluntary and self-reported; therefore, the survey results may be biased due to inaccurate self-reporting, nonresponse, and selection bias. There may be an overrepresentation of participants familiar with ChatGPT. Due to the different structure of medical education internationally, the results may be less generalizable outside of the United States. Another limitation is that we limited responses on use of ChatGPT to those who had been previously reported in the literature; our survey did not include free-response items, and thus, we may have missed novel potential applications of ChatGPT for academic, clinical, or research purposes that students are using it for.

### Implications

Future directions include examining resident and attending physicians’ use of AI tools, such as LLMs, to understand the attitudes and perceptions of those currently providing direct clinical care. A cross-sectional study by the Prince of Songkla University surveying both medical students and physicians found that graduated physicians tended to have less favorable perceptions of ChatGPT for clinical practice and medical education compared to medical students [4]. This may reflect that LLMs are less clinically useful as physicians develop more expertise, as participants listed concerns that ChatGPT lacks patient-specific treatment plans, provides superficial responses, and does not incorporate updated evidence [4]. As AI models evolve, with use specifically tailored toward medical queries, it will be interesting to examine how clinicians’ perspectives might change.

### Conclusions

This study highlights the current adoption of ChatGPT among medical students throughout the United States and underscores its perceived utility in academic, clinical, and research settings. A significant majority of students in our survey reported using ChatGPT to enhance their learning experience, with notable use cases including understanding complex medical concepts, preparing for exams, and generating study materials. Clinical and research applications were also prevalent, particularly in generating differential diagnoses, reviewing pharmacological information, and summarizing research papers.

Despite its benefits, our findings raise concerns regarding information verification, ethical use for academic activities, and AI literacy. A substantial proportion of students reported infrequent cross-checking or editing of ChatGPT-generated responses, which poses a real risk of misinformation in medical training. This emphasizes the need for improved education on AI’s limitations and potential biases. Notably, students with a higher baseline understanding of AI were more likely to verify and refine ChatGPT’s output, reinforcing the importance of integrating AI education into medical training. Moreover, the use of ChatGPT for completing assignments may undermine learning outcomes, highlighting the need for updated academic integrity policies in medical education. Finally, the use of ChatGPT in patient care also raises HIPAA-compliance concerns, further emphasizing the need for updated policies to deliver the safest and most effective patient care.

Given the rapid evolution of AI-powered tools, medical educators should consider incorporating structured AI literacy programs to equip students with the skills necessary to use these technologies responsibly and effectively. Guidance on the use of AI in medical schools may be further standardized by collaborating with national accrediting bodies and medical education organizations. Ultimately, more research is needed to explore best practices for AI integration in medical education and to assess the long-term impact of AI-assisted learning on clinical decision-making and patient care.

## Appendix

Multimedia Appendix 1 Survey questionnaire.

## Abbreviations

AI: artificial intelligence
CHERRIES: Checklist for Reporting Results of Internet E-Surveys
HIPAA: Health Insurance Portability and Accountability Act
LLM: large language model
OR: odds ratio

## Data Availability

The datasets generated or analyzed during this study are available from the corresponding author on reasonable request.

## References

[1] Ghassemi M, Birhane A, Bilal M, Kankaria S, Malone C, Mollick E, Tustumi F (2023). ChatGPT one year on: who is using it, how and why?. Nature.

[2] Timothy M (2023). 5 Reasons why ChatGPT became the fastest growing app of all time. Make Use Of.

[3] Bin-Nashwan SA, Sadallah M, Bouteraa M (2023). Use of ChatGPT in academia: academic integrity hangs in the balance. Technol Soc.

[4] Tangadulrat P, Sono S, Tangtrakulwanich B (2023). Using ChatGPT for clinical practice and medical education: cross-sectional survey of medical students' and physicians' perceptions. JMIR Med Educ.

[5] Weidener L, Fischer M (2024). Artificial intelligence in medicine: cross-sectional study among medical students on application, education, and ethical aspects. JMIR Med Educ.

[6] Abu Hammour A, Hammour KA, Alhamad H, Nassar R, El-Dahiyat F, Sawaqed M, Allan A, Manaseer Q, Abu Hammour M, Halboup A, Farha RA (2024). Exploring Jordanian medical students' perceptions and concerns about ChatGPT in medical education: a cross-sectional study. J Pharm Policy Pract.

[7] Xu AY, Piranio VS, Speakman S, Rosen CD, Lu S, Lamprecht C, Medina RE, Corrielus M, Griffin IT, Chatham CE, Abchee NJ, Stribling D, Huynh PB, Harrell H, Shickel B, Brennan M (2024). A pilot study of medical student opinions on large language models. Cureus.

[8] Ganjavi C, Eppler M, O'Brien D, Ramacciotti LS, Ghauri MS, Anderson I, Choi J, Dwyer D, Stephens C, Shi V, Ebert M, Derby M, Yazdi B, Cacciamani GE (2024). ChatGPT and large language models (LLMs) awareness and use. A prospective cross-sectional survey of U.S. medical students. PLOS Digit Health.

[9] Ichikawa T, Olsen E, Vinod A, Glenn N, Hanna K, Lund GC, Pierce-Talsma S (2025). Generative artificial intelligence in medical education-policies and training at US osteopathic medical schools: descriptive cross-sectional survey. JMIR Med Educ.

[10] Eysenbach G (2004). Improving the quality of Web surveys: the Checklist for Reporting Results of Internet E-Surveys (CHERRIES). J Med Internet Res.

[11] Quantitative research design (JARS–Quant). American Psychological Association.

[12] Xu X, Chen Y, Miao J (2024). Opportunities, challenges, and future directions of large language models, including ChatGPT in medical education: a systematic scoping review. J Educ Eval Health Prof.

[13] Miftahul Amri M, Khairatun Hisan U (2023). Incorporating AI tools into medical education: harnessing the benefits of ChatGPT and Dall-E. J Nov Eng Sci Technol.

[14] Hirosawa T, Kawamura R, Harada Y, Mizuta K, Tokumasu K, Kaji Y, Suzuki T, Shimizu T (2023). ChatGPT-generated differential diagnosis lists for complex case-derived clinical vignettes: diagnostic accuracy evaluation. JMIR Med Inform.

[15] Shah-Mohammadi F, Finkelstein J (2024). Accuracy evaluation of GPT-assisted differential diagnosis in emergency department. Diagnostics (Basel).

[16] Shea YF, Lee CM, Ip WC, Luk DW, Wong SS (2023). Use of GPT-4 to analyze medical records of patients with extensive investigations and delayed diagnosis. JAMA Netw Open.

[17] Bhattacharyya M, Miller VM, Bhattacharyya D, Miller LE (2023). High rates of fabricated and inaccurate references in ChatGPT-generated medical content. Cureus.

[18] Cascella M, Montomoli J, Bellini V, Bignami E (2023). Evaluating the feasibility of ChatGPT in healthcare: an analysis of multiple clinical and research scenarios. J Med Syst.

[19] Eysenbach G (2023). The role of ChatGPT, generative language models, and artificial intelligence in medical education: a conversation with ChatGPT and a call for papers. JMIR Med Educ.

[20] Alkaissi H, McFarlane SI (2023). Artificial hallucinations in ChatGPT: implications in scientific writing. Cureus.

[21] Alkhaaldi SM, Kassab CH, Dimassi Z, Oyoun Alsoud L, Al Fahim M, Al Hageh C, Ibrahim H (2023). Medical student experiences and perceptions of ChatGPT and artificial intelligence: cross-sectional study. JMIR Med Educ.

[22] Magalhães Araujo S, Cruz-Correia R (2024). Incorporating ChatGPT in medical informatics education: mixed methods study on student perceptions and experiential integration proposals. JMIR Med Educ.

[23] Grava-Gubins I, Scott S (2008). Effects of various methodologic strategies: survey response rates among Canadian physicians and physicians-in-training. Can Fam Physician.

[24] Tater J, Zaharic T, Guy W, Cornwall J (2023). How much is too much? Medical students’ perceptions of evaluation and research requests, and suggestions for practice. Assess Eval High Educ.

