# Supplementary material for: Medical Student Experiences With ChatGPT: National Cross-Sectional Study
Source: JMIR Form Res. 2026 Mar 9;10:e76838. doi: 10.2196/76838 (PMC13010067; doi:10.2196/76838)
Supplement: Supplementary file 1 [file formative_v10i1e76838_app1.docx]

FINAL EDITS - Medical Student Perceptions of ChatGPT

Start of Block: Artificial Intelligence Baseline Proficiency and Usage

	
		
		
	
	
		
			
			Title of Project: 
			
			
			

			U.S. Medical Student Opinions on Artificial Intelligence
			
		
		
			
			Principal Investigator:
			
			
			Meghan M Brennan, MD, MS
			
		
	

 

Please read the information below carefully before you decide to participate in this research study. Your participation is voluntary.  You can decide not to participate or later decide to stop participating at any time without penalty or lose any benefits that would normally be expected.

 

	
	Purpose of the Study: The purpose of this research study is to assess U.S. medical students' current usage of artificial intelligence (AI) in medical school, their opinion of this technology, and their predictions about its future use in medicine. 
	

	
	What will you be asked to do: You will be asked to answer questions about your usage and opinion of AI. You will also be asked to provide your demographic information.
	

	
	Time Required:  It will take about 5 minutes to participate in the research.
	

	
	Research Benefits:  There are no direct benefits to you for being in this study. There may be a benefit to others depending on the results of this study.
	

	
	Research Risks: There are no risks or discomforts anticipated. Your responses will be anonymous and confidential. Responses will be published as aggregate, not individual, data.
	

	
	Statement of Confidentiality: Your participation in this research is confidential. Information collected about you will be stored in computers with security passwords or in locked filing cabinets. Only certain people have the legal right to review these research records, and they will protect the secrecy (confidentiality) of these records as much as the law allows. These people include the researchers for this study, certain University of Florida officials, and the Institutional Review Board (IRB; an IRB is a group of people who are responsible for looking after the rights and welfare of people taking part in research). Otherwise your research records will not be released without your permission unless required by law or a court order.  The researchers will not share your name or other identifiable information about you if they publish, present, or share the results of this research.     
	

	
	Who to contact if you have questions: Please contact Dr. Meghan M. Brennan at mbrennan@anest.ufl.edu or Alan Xu at axu@ufl.edu with questions or concerns about this study. 
	

	
	Voluntary Participation: Your decision to be in this research is voluntary. You do not have to do any study activities that you do not want to take part in.  You can stop at any time. If you decide you want to stop participating in the research, you can let the research team know or contact the Principal Investigator at any time at mbrennan@anest.ufl.edu.   If you choose not to take part, this will have no effect on you or your relationships with the University of Florida.  If you have any questions about your rights as a research subject, you can phone the Institutional Review Board at 352-273-9600.
	

Participation in the research implies that you have read the information in this form and consent to take part in the research. Please save a copy of this form for your records or future reference.

If you want to participate in this research study, click the “I agree to participate” button below.  If you do not want to participate, you may simply close this window.

- I agree to participate  (4) 

| Page Break |  |
| --- | --- |

How would you describe your current understanding of artificial intelligence (AI)?

- **No understanding**: I have never heard of AI.  (6) 

- **Limited understanding**: I have heard of the terminology but do not understand how it works.  (1) 

- **Basic understanding**: I understand that AI involves machines performing tasks that typically require human intelligence, but I'm not familiar with its applications.  (2) 

- **Moderate understanding**: I have a general understanding of the key principles of AI and its common applications.  (3) 

- **Advanced understanding**: I possess a comprehensive understanding of AI principles, including deep learning, neural networks, and their complex implementations in real-world scenarios.  (5) 

Have you ever heard of ChatGPT (a chatbot and virtual assistant developed by OpenAI)?

- Yes  (7) 

- No  (8) 

Have you ever used ChatGPT?

- Yes  (27) 

- No  (28) 

End of Block: Artificial Intelligence Baseline Proficiency and Usage

Start of Block: Frequency of ChatGPT Usage - Academic

|  |
| --- |

Have you used ChatGPT for the following **academic purposes** in medical school?

|  | Yes (33) | No (34) |
| --- | --- | --- |
| Understanding complex medical concepts or terminology (1) |  |  |
| Generating study materials such as summaries or outlines (2) |  |  |
| Completing school assignments (3) |  |  |
| Creating practice questions to test yourself (8) |  |  |
| Simulating patient interviews or scenarios (9) |  |  |
| Preparing for exams or quizzes (10) |  |  |

End of Block: Frequency of ChatGPT Usage - Academic

Start of Block: Frequency of ChatGPT Usage - Clinical

|  |
| --- |

Have you used ChatGPT for the following **clinical or patient care** **purposes** in medical school?

|  | Yes (34) | No (35) |
| --- | --- | --- |
| Generating differential diagnoses (10) |  |  |
| Interpreting lab results (3) |  |  |
| Providing patient education materials (4) |  |  |
| Writing clinical notes or summaries (1) |  |  |
| Researching clinical guidelines (8) |  |  |
| Formulating management or treatment plans (6) |  |  |
| Reviewing pharmacological information (12) |  |  |
| Preparing for patient encounters (e.g. questions to ask) (15) |  |  |
| Developing strategies for handling complex or challenging patient scenarios (16) |  |  |

End of Block: Frequency of ChatGPT Usage - Clinical

Start of Block: Frequency of ChatGPT Usage - Research

|  |
| --- |

Have you used ChatGPT for the following **research purposes** in medical school?

|  | Yes (29) | No (30) |
| --- | --- | --- |
| Formulating research questions or hypotheses (2) |  |  |
| Summarizing research articles or studies (3) |  |  |
| Analyzing research data (4) |  |  |
| Drafting research papers or reports (5) |  |  |
| Reviewing statistical methods or analyses (6) |  |  |
| Exploring recent medical research trends (8) |  |  |

End of Block: Frequency of ChatGPT Usage - Research

Start of Block: Perception of ChatGPT

When utilizing ChatGPT for medical-related queries, **how would you rate the accuracy of information given**?

- **Very inaccurate**: consistently gives incorrect information  (1) 

- **Inaccurate**: more often gives incorrect information  (2) 

- **Somewhat inaccurate/accurate:** gives similar frequency of incorrect as correct information  (8) 

- **Accurate**: more often gives correct information  (4) 

- **Very accurate**: consistently gives correct information  (5) 

- N/A; I have not used ChatGPT in this context  (9) 

When utilizing ChatGPT for medical school-related queries, how often do you **cross-check or double-check the responses you receive**?

- **Almost never**: about 0% of the time  (1) 

- **Occasionally**: about 25% of the time  (2) 

- **Sometimes**: about 50% of the time  (4) 

- **Frequently**: about 75% of the time  (7) 

- **Almost always**: almost 100% of the time  (5) 

- N/A; I have not used ChatGPT in this context  (9) 

When utilizing ChatGPT for medical school-related purposes, how often do you **edit or refine ChatGPT's output before using it**?

- **Almost never**: about 0% of the time  (1) 

- **Occasionally**: about 25% of the time  (2) 

- **Sometimes**: about 50% of the time  (4) 

- **Frequently**: about 75% of the time  (7) 

- **Almost always**: almost 100% of the time  (5) 

- N/A; I have not used ChatGPT in this context  (9) 

Overall, the responses provided by ChatGPT were useful for your medical school-related queries

- Strongly disagree  (1) 

- Disagree  (2) 

- Agree  (4) 

- Strongly Agree  (3) 

- N/A; I have not used ChatGPT in this context  (5) 

Do you believe you will use ChatGPT or similar AI chatbots in your *future* medical practice (e.g. during rotations or as a physician)?

- Yes  (29) 

- No  (30) 

How has your experience with ChatGPT influenced your overall perception of AI’s potential to assist in medical practice?

- Significantly worsened  (28) 

- Somewhat worsened  (29) 

- No change  (30) 

- Somewhat improved  (31) 

- Significantly improved  (32) 

To what extent do you agree that **having ChatGPT as a resource would help you better manage patient care and make you a more effective doctor**?

- Strongly disagree  (1) 

- Disagree  (2) 

- Agree  (4) 

- Strongly Agree  (5) 

End of Block: Perception of ChatGPT

Start of Block: Implications of Artificial Intelligence 

Right now, I trust artificial intelligence (AI) to assist in clinical decision-making:

- Strongly disagree  (1) 

- Disagree  (2) 

- Agree  (4) 

- Strongly agree  (5) 

In the next 5 years, I anticipate trusting AI to assist in clinical decision-making:

- Strongly disagree  (1) 

- Disagree  (2) 

- Agree  (4) 

- Strongly agree  (5) 

Have you been exposed to any AI integration or education in your medical school's curriculum?

- Yes  (9) 

- No  (10) 

Do you believe concepts of AI should be taught in medical school?

- Yes  (8) 

- No  (9) 

|  |
| --- |

I would feel comfortable with AI assisting with:

|  | Agree (1) | Disagree (2) |
| --- | --- | --- |
| Writing medical notes. (1) |  |  |
| Interpreting medical imaging scans. (7) |  |  |
| Interpreting lab test results. (19) |  |  |
| Diagnosing patients. (2) |  |  |
| Creating treatment plans for patients. (10) |  |  |
| Completing administrative tasks such as handling billing and scheduling appointments. (5) |  |  |
| Communicating with patients such as interpreting medical information. (13) |  |  |
| Organizing the flow of a patient visit. (20) |  |  |

|  |
| --- |

I believe the use of AI in healthcare will...

|  | Agree (1) | Disagree (2) |
| --- | --- | --- |
| Expand access to healthcare services. (1) |  |  |
| Reduce clinical reasoning skills. (13) |  |  |
| Reduce physician decision-making autonomy. (8) |  |  |
| Lead to a reduction in physician jobs. (9) |  |  |
| Lead to issues with accountability for medical errors. (10) |  |  |
| Reduce time spent on administrative tasks. (14) |  |  |
| Enhance the speed and accuracy of diagnoses and treatment. (16) |  |  |
| Enhance the overall quality of patient care. (17) |  |  |

End of Block: Implications of Artificial Intelligence 

Start of Block: Demographics

What is your sex?

- Male  (1) 

- Female  (2) 

- Other  (3) 

- Prefer not to answer  (5) 

What is your age?

- Under 20  (1) 

- 20-24  (2) 

- 25-30  (3) 

- Over 30  (4) 

How do you self-identify?

- White  (9) 

- Black or African American  (10) 

- American Indian or Alaska Native  (11) 

- Hispanic, Latino, or of Spanish origin  (16) 

- Asian  (12) 

- Native Hawaiian or other Pacific Islander  (13) 

- Other  (14) 

- Prefer not to answer  (15) 

What is the name of the medical school you attend (optional)?

________________________________________________________________

In what year of medical school are you? Note: Students in dual degree programs (e.g. MD-PhD) who are not actively in a medical school year should select highest year of medical school completed.

- First year  (1) 

- Second year  (2) 

- Third year  (3) 

- Fourth year  (4) 

- Fifth year or beyond  (5) 

Have you completed at least one block of clinical rotations? (Traditionally, these begin during the 3rd year of medical school)

- Yes  (8) 

- No  (9) 

What medical residency are you most interested in pursuing currently?

▼ Unsure (1) ... Other (31)

End of Block: Demographics
